# Supplementary material for: Multi-compartmental model of glymphatic clearance of solutes in brain tissue
Source: PLoS One. 2023 Mar 7;18(3):e0280501. doi: 10.1371/journal.pone.0280501 (PMC9990927; doi:10.1371/journal.pone.0280501)
Supplement: S1 Appendix — (PDF) [file pone.0280501.s001.pdf]

S1 Appendix

A Computing biologically relevant parameters

A.1 Permeability coefficients

In the present article, we use a definition of the permeability coefficients that can be obtained from the resistance values given in [48]. Even though this latter work considers a one-dimensional model, a relation between 1D resistances and 3D permeabilities can be found. Indeed, assuming that a 1D line is embedded into a 3D cylinder of length L and cross-sectional area A, the volumetric flux in the 1D geometry is given by the Poiseuille equation

$$Q = \frac{1}{R}\Delta p,$$

where R is the resistance in the 1D geometry and Δp is the pressure difference between the two ends of the line. Then, if we assume that the flow in the 3D cylinder is given only by the flow in the line, Darcy's law gives the relation

$$Q = \frac{\kappa}{\mu}\frac{A}{L}\Delta p = \frac{1}{R}\Delta p,$$

where κ is the averaged permeability and μ is the dynamic viscosity. Altogether, we obtain

$$\kappa = \frac{\mu L}{RA}. \tag{20}$$

For all compartments, $\frac{L}{A}$ is related to the length scale of the brain. Therefore, knowing the permeability of the ECS, for example, from [2] and the resistance as computed by [48], we obtain a constant relationship between R_j and κ_j for all compartments. Resistance coefficients R_j for the different compartments can be found in [48]. Choosing a permeability for the ECS of $\kappa_e = 2.0 \times 10^{-11}\text{mm}^2$, a CSF dynamic viscosity of $\mu_e = 0.7 \times 10^{-3}$ Pa s, and the resistance coefficient $R_e = 4.56(\text{Pa s})\text{mm}^3$ indicated in [48] we have

$$\frac{L}{A} = 1.3 \times 10^{-7}\ \text{mm}^{-1},$$

and we obtain the values for the permeability coefficients

$$\kappa_{pa} = 1.0 \times 10^{-11}\ \text{mm}^2, \kappa_{pv} = 6.51 \times 10^{-9}\ \text{mm}^2, \kappa_{pc} = 3.54 \times 10^{-13}\ \text{mm}^2.$$

Then, from [49] and [50], as well as choosing a dynamic viscosity of blood $\mu_{\text{blood}} = 2.67 \times 10^{-3}$ Pa s [29]

$$\kappa_a = 3.30 \times 10^{-6}\ \text{mm}^2, \kappa_v = 6.59 \times 10^{-6}\ \text{mm}^2, \kappa_c = 8.8 \times 10^{-9}\ \text{mm}^2.$$

It is worth mentioning that in previous works related to multi-compartment modelling of the glymphatic system, several authors evaluated these coefficients through numerical testing, leading to very different values. Indeed, from [29, 32, 93] in which the MPET equations are used to represent the movement of CSF through different compartments in the brain, permeabilities are

$$\kappa_a = \kappa_v = \kappa_c = \kappa_{pa} = \kappa_{pv} = \kappa_{pc} = 1.0 \times 10^{-4}\ \text{mm}^2 \kappa_e = 1.4 \times 10^{-8}\ \text{mm}^2.$$

Therefore, we obtain a difference between these two parameter sets of several orders of magnitude, leading to tremendous differences in fluid movement.

A.2 Transfer coefficients

Following the Starling equation, the definition of the coefficients for transfer between vessels and tissues is

$$\gamma_{i,j} = L_{i,j}\frac{|S_{i,j}|}{|\Omega|}, \tag{21}$$

where $L_{i,j}$ is the hydraulic conductivity of the membrane (in mm/(s PA)), $\frac{|S_{i,j}|}{|\Omega|}$ is the ratio between the surface area of the vessel per unit of volume of tissue (in mm^{-1}).

We know the ratio $\frac{|S_{i,j}|}{|\Omega|}$, but we are missing the value of the hydraulic conductivity for some of the considered membranes. For the transfer from blood vessels to ECS, we can find the value of the hydraulic conductivity of the BBB at the different levels (*i.e.* arteries, capillaries and veins). These values are reported in the main body of this article, in Section 2.

For the transfer coefficients between PVSs and the ECS, we use the following method. We search for a suitable relation between the 1D resistance parameters from [48] and the 3D exchange coefficients $\gamma_{j,i}$. In the following, we assume that the transfer coefficients for the PVSs to ECS are comparable between humans and rats.

Starting from the volumetric flow $Q_{j,i}$ through a 1D structure

$$Q_{j,i} = \frac{1}{R_{j,i}}(p_i - p_j),$$

where $R_{j,i}$ is the resistance through the structure and using the fact that this same volumetric flux in 3D is given by

$$Q_{j,i} = \int_{\Omega}\gamma_{j,i}(p_i - p_j)\,\mathrm{d}x,$$

assuming that the pressure difference $(p_i–p_j)(x)$ is constant in space (which is not unreasonable since the transfer coefficient is homogeneous in space as well), we obtain the relation

$$\gamma_{j,i} = \frac{1}{R_{j,i}|\Omega|}.$$

We emphasize that since the resistance coefficients reported here are for humans, the volume $|\Omega|$ is the volume of the human brain, *i.e.* $|\Omega| = 1 \times 10^6 \mathrm{mm}^3$. Thus, from this equation, we can define the transfer coefficient in a different manner using only the 1D resistances estimated in [48] and the volume of our computational domain.

We apply the previously presented method to compute the exchange coefficients between PVSs and ECS. We obtain

$$\gamma_{pa,e} = 2.19 \times 10^{-7}\,(\mathrm{Pa\ s})^{-1}, \gamma_{pv,e} = 1.95 \times 10^{-7}\,(\mathrm{Pa\ s})^{-1}, \gamma_{pc,e} = 9.19 \times 10^{-9}\,(\mathrm{Pa\ s})^{-1}.$$

For the exchange from blood vessels to ECS, we obtain

$$\gamma_{a,e} = 2.73 \times 10^{-9}\,(\mathrm{Pa\ s})^{-1}, \quad \gamma_{v,e} = 6.00 \times 10^{-11}\,(\mathrm{Pa\ s})^{-1}, \quad \gamma_{c,e} = 9.00 \times 10^{-10}\,(\mathrm{Pa\ s})^{-1}.$$

Next, we need to specify the transfer coefficients for connected spaces, *e.g.* from arteries to capillaries. To do so, we use the equation

$$\gamma_{j,i} = \frac{|q|}{|\Delta p_{i,j}|},$$

where Q is the flow rate of fluid (CSF or blood) and $\Delta p_{i,j}$ denotes the pressure drop from one compartment to the other. Using values from Table 7 and using a value of 2.0g for the weight of the brain (see [97]) as well as a pressure drop from arteries to capillaries of $\Delta p_{a,c}$ = 40mmHg, and a pressure drop from capillaries to veins of $\Delta p_{c,v}$ = 13mmHg, we obtain

$$\gamma_{a,c} = 3.14 \times 10^{-6}\,(\text{Pa s})^{-1}, \quad \gamma_{c,v} = 9.65 \times 10^{-6}\,(\text{Pa s})^{-1}.$$

Then, assuming a total flow rate of CSF through perivascular spaces of Q_{CSF} = 3.38μL/min (which corresponds to CSF production rate, see [60], and clearly represents an upper estimate of the actual flow in the PVS), and a pressure drop from PVS arteries to PVS capillaries of $\Delta p_{pa,pc}$ = 1mmHg, and a pressure drop from PVS capillaries to PVS veins of$\Delta p_{pc,pv}$ = 0.25mmHg (both of these latter values are assumed to be correct but we emphasize that we could not find any measurement in the literature), we obtain

Table 7. Blood and CSF parameters.

Name	Unit	Value	Reference
Cerebral blood flow (CBF)	mL/g/min	1.16	[58]
CSF production rate	μL/min	3.38	[59]
Mean arterial blood pressure (MAP)	mmHg	95	[94]
Pial venous pressure	mmHg	7	[95]
Pial arteriolar pressure	mmHg	56	[95, 96]

$$\gamma_{pa,pc} = 1.83 \times 10^{-7}\,(\text{Pa s})^{-1}, \quad \gamma_{pc,pv} = 7.31 \times 10^{-7}\,(\text{Pa s})^{-1}.$$

The coefficients $\tilde{\gamma}_{j,i}$ are given by the value of the reflection coefficient $\sigma_{\text{reflect},ij}$ and the equation

$$\tilde{\gamma}^{14\text{C-inulin}}{}_{j,i} = \gamma_{j,i}(1 - \sigma^{14\text{C-inulin}}_{\text{reflect},ij}). \tag{22}$$

We also define the hydraulic permeability of the fluid at the pial surface to define the Robin boundary conditions. Therefore, we search the 3D coefficients $\gamma_{i,j}$ using the previous method, and we then compute the hydraulic conductivity $L_{i,j}$ that we can use in the definition of the boundary conditions. We assume that the boundary permeability for the ECS compartment is given by a resistance coefficient that we assume to be twice larger than the resistance coefficient of the PVS of arteries, *i.e.* $R_{e,SAS} = 2 \times R_{pa}$ (we emphasize again that we could not find a measurement of this hydraulic conductivity at the pial surface of the brain). Then, using the relation

$$L_{i,j} = \frac{1}{R_{i,j}|S_{i,j}|},$$

where $|S_{i,j}|$ corresponds to the surface area of the pial membrane of the human brain ($\approx$1750 $\times$ 10^2 mm^2), we obtain

$$L_{e,\mathrm{SAS}} = 3.13 \times 10^{-7}\,\mathrm{mm/(Pa\ \ s)}, \quad L_{pa,\mathrm{SAS}} = 1.25 \times 10^{-6}\,\mathrm{mm/(Pa\ \ s)}.$$

The next coefficient to define is $\lambda_{i,j}$ for the mass transfer of the solute. Following the definition of diffusive mass transfer, we know that

$$\lambda_{i,j} = P_{i,j} \frac{A_{\mathrm{vessel}}}{V_{\mathrm{tissue}}}, \tag{23}$$

where $P_{i,j}$ is the permeability (in mm/s) between the two compartments.

The diffusive permeabilities are computed using the method from [63], namely for the permeability to the molecule $\alpha = {}^{14}\mathrm{C}$-inulin, we have

$$P^{\alpha} = \frac{1}{\pi D_v} \sum_{r \in F} \frac{1}{R_r^{\alpha}},$$

where D_v corresponds to the diameter of the considered vessel (10×10^{-3} mm for capillaries [98], 50×10^{-3} for arterioles and venules [99, 100]), F is the index set corresponding to the different layers of the membrane for which we compute the permeability, R_r^{α} is the resistances

to solute transport for the different layers. For the AEF barrier, the only layer to cross is the astrocyte endfeet processes. We have the definition of the resistance

$$R_{\mathrm{AEF}} = \frac{L_{\mathrm{AEF}}}{2 B_{\mathrm{AEF}} D_{\mathrm{AEF}}^{\alpha}},$$

where L_{AEF} is the width of the membrane, $2B_{\mathrm{AEF}}$ is the width between two astrocyte endfeet (at the perivenous and periarterial level, we take B_{AEF} = 250nm and at the pericapillary level, we take B_{AEF} = 25nm), and $D_{\mathrm{AEF}}^{\alpha}$ is the diffusion coefficient in this same cleft. Assuming that the cleft has a cylindrical shape, the latter parameter is assumed to be given from the relation [64]

$$\begin{cases} D_{\mathrm{AEF}}^{\alpha} = D_{\mathrm{free}}^{\alpha}(1 - 2.10444\beta + 2.08877\beta^3 - 0.094813\beta^5 - 1.372\beta^6), \\ \beta = \dfrac{a^{\alpha}}{B_{\mathrm{AEF}}}, \end{cases}$$

in which a^{α} is the solute radius. The Stokes radius of inulin is indicated to be $a^{\mathrm{Inulin}} = 15.2 \times 10^{-7}$ mm in [101]. Finally, we obtain

$$\lambda_{pa,e}^{^{14}\mathrm{C-inulin}} = 3.70 \times 10^{-3}\,\mathrm{s}^{-1}, \quad \lambda_{pv,e}^{^{14}\mathrm{C-inulin}} = 3.72 \times 10^{-3}\,\mathrm{s}^{-1}, \quad \lambda_{pc,e}^{^{14}\mathrm{C-inulin}} = 3.70 \times 10^{-3}\,\mathrm{s}^{-1}.$$

A.3 Variations of PVS porosities

In our article, we assumed some variations of the PVSs volume. Using the resistance formula provided in [48] which gives

$$R \propto \frac{1}{r_1^4},$$

where r_1 is the inner radius of the PVS. Thus, with our equation for the permeability coefficient (20), we obtain the proportionality relation

$$\kappa_j \propto r_1^4.$$

Furthermore, assuming that the PVSs are just holed cylinders, the change of volume is proportional to the change in r_1^2. Therefore, from the two previous proportionality relations, we obtain that multiplying the volume of the PVS by a constant C results in multiplying the permeability by the square of this constant.
