# Supplementary material for: Multi-compartmental model of glymphatic clearance of solutes in brain tissue
Source: PLoS One. 2023 Mar 7;18(3):e0280501. doi: 10.1371/journal.pone.0280501 (PMC9990927; doi:10.1371/journal.pone.0280501)
Supplement: S2 Appendix — (PDF) [file pone.0280501.s002.pdf]

S2 Appendix

B Sensitivity analysis

Fig 8 shows the model sensitivity to parameter changes in the 4-compartment model. The largest effect is seen for changes in diffusion coefficient. The corresponding sensitivity to parameters for the 7-compartment model is shown in Fig 9. The results are still affected by changes in the diffusion coefficient, but the greatest effect was seen from changes in the permeability for fluid to be secreted from arteries to periarterial spaces.

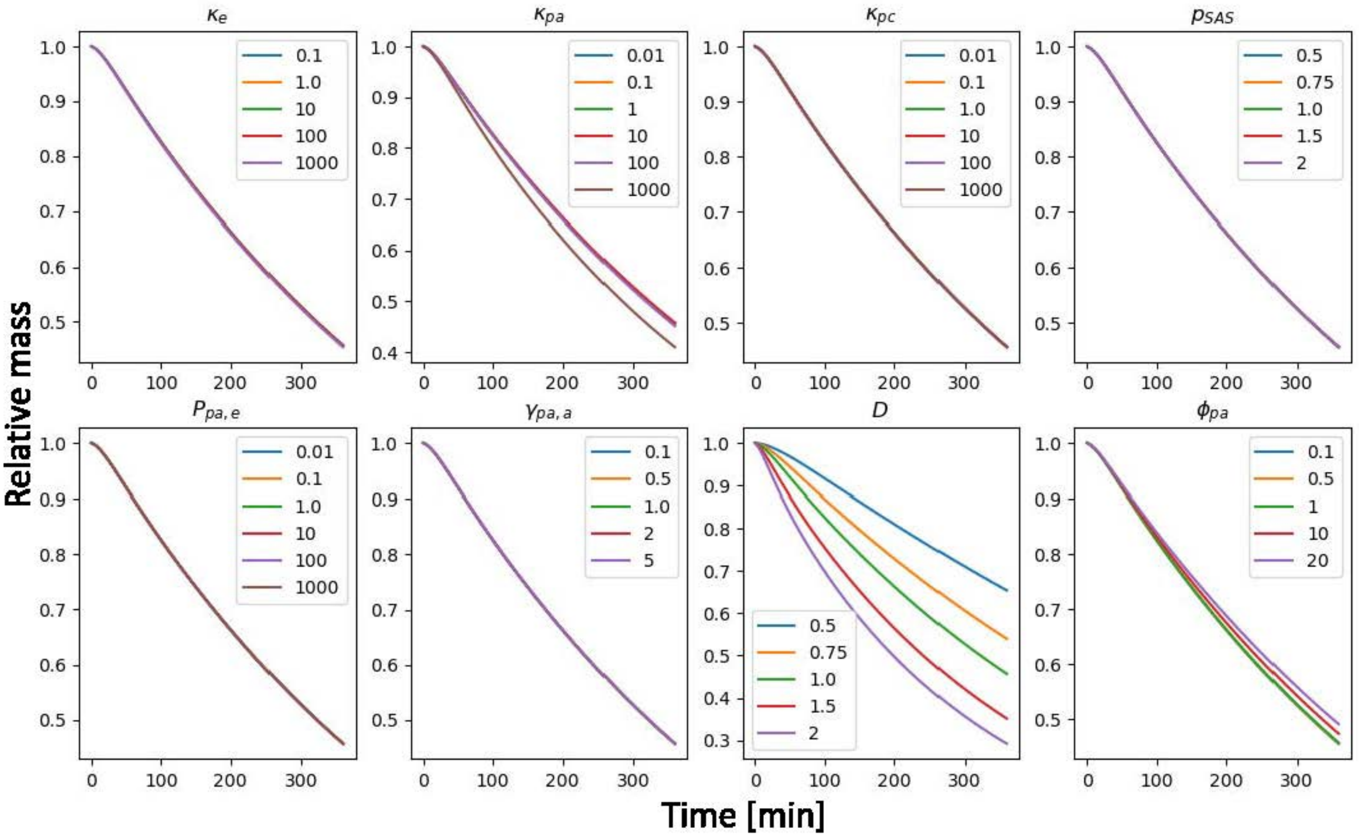

Fig 8. Sensitivity analysis of the 4-compartment model. Each curve represents a change in the parameter of a given factor. The largest effects were seen in changes in the periarterial permeability, the diffusion coefficient and the periarterial porosity.

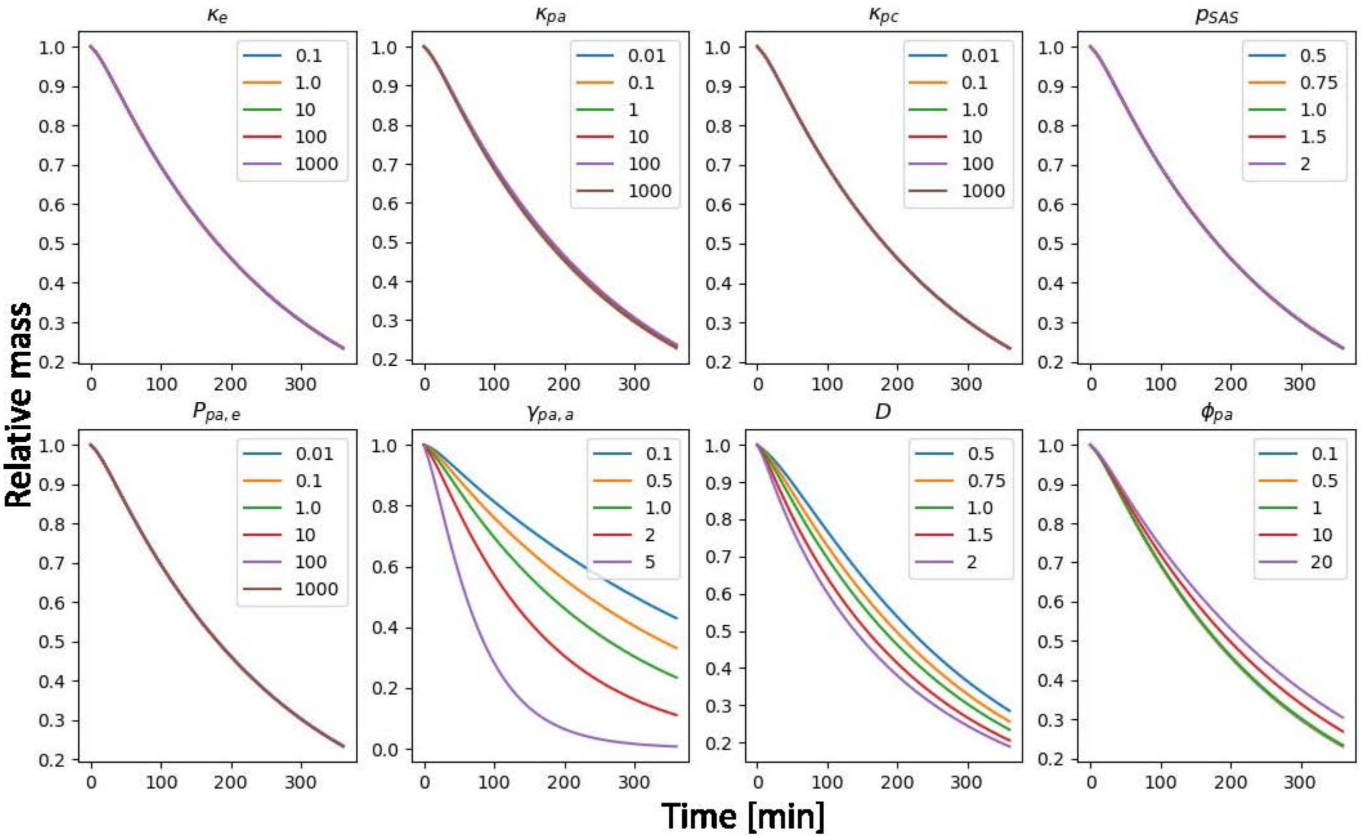

Fig 9. Sensitivity analysis of the 7-compartment model. Each curve represents a change in the parameter of a given factor. The undisputed greatest effect was seen in changes in the convective fluid transfer permeability between the periarterial and arterial networks.
