# Supplementary material for: Multi-compartmental model of glymphatic clearance of solutes in brain tissue
Source: PLoS One. 2023 Mar 7;18(3):e0280501. doi: 10.1371/journal.pone.0280501 (PMC9990927; doi:10.1371/journal.pone.0280501)
Supplement: S3 Appendix — (PDF) [file pone.0280501.s003.pdf]

## S3 Appendix

# C Numerical verification

## C.1 Method of manufactured solutions

To ensure the correctness of the implemented numerical solver, we use the method of manufactured solutions. Consider the square $\Omega = [-1, 1]^2$, and define the functions

$$p_j(x, y) = a_j \cos(\pi x/2)\cos(\pi y/2) + p_j^0, \quad c_j(x, y, t) = b_j(1 - t/T)(x^2 + y^2) + c_j^0 \tag{24}$$

for $j \in \{e, pa, pc, pv\}$, where $a_j$, $b_j$, $p_j^0$ and $c_j^0$ are some predetermined constants and $T$ is the end time of the simulations. For these functions to be valid solutions, we need to augment each of the modelling equations by an additional source term, chosen such that the functions defined in (24) solve the problem. Since the model with the additional source terms defines a more general form of our original problem, then a solution algorithm for the multi-compartment model *with* sources should be able to solve the problem *without* the source terms as well (which is equivalent to setting each of the sources to $f_j$).

Fig 10 plots the errors of the numerically obtained solutions compared to the analytically correct solutions defined in (24) for varying mesh resolution. Denoting by $V = H^1(\Omega)^{|J|}$, where $|J| = 4$ is the number of compartments in the model, the error for the pressure equations is measured in the norm

$$\|u\|_V = \sqrt{\sum_j \|u_j\|^2_{H^1(\Omega)}}, \tag{25}$$

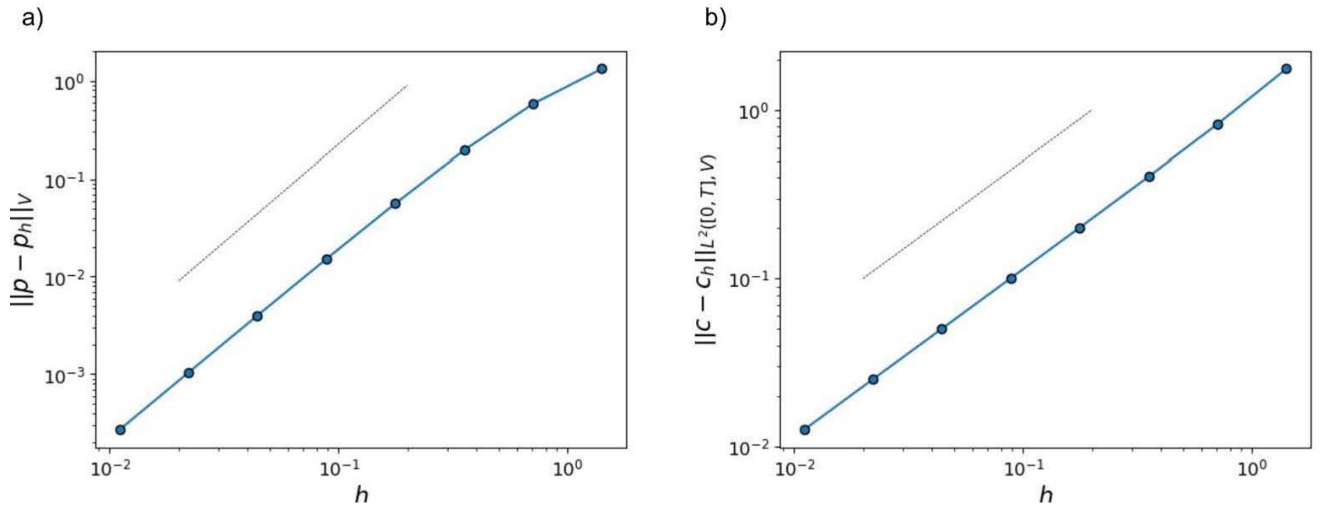


**Fig 10. Error of the numerical solution for varying mesh resolution, supplemented by a dashed black line indicating the expected convergence rate.** a) Convergence plot for the pressure equation. The error converges quadratically with respect to cell size, measured in the norm defined in (25). b) Convergence plot for the concentration equation. The error converges linearly with respect to cell size, measured in the Bochner-space norm (26).

The time-dependent concentration equations error for they are measured in the following approximate Bochner-space norm,

$$\|u\|_{L^2([0,T],V)} = \sqrt{\int_0^T \|u(t)\|_V^2 \, dt} \approx \sqrt{\sum_{n=0}^{N-1} \frac{\Delta t}{2}\left(\|u^n\|_V^2 + \|u^{n+1}\|_V^2\right)}, \tag{26}$$

where $u^n$, $n = 0, \ldots, N$ is the numerical solution at time $t_n$.

As shown in Fig 10a, the solver for the pressure equations exhibits quadratic convergence with respect to the largest cell size $h_{\max}$, as expected from the Bramble-Hilbert lemma applied to piecewise linear elements on a shape-regular triangulation [102, p. 79 Theorem 6.4]. Similarly, Fig 10b shows a linear error convergence with respect to the cell size, as expected from e.g. [103, Theorem 5.1 p. 134].

These results verify the correctness of the implemented numerical solver and that the baseline parameters do not introduce any significant numerical challenges. We can not, however, exclude that some numerical issues are introduced when going to the complex three-dimensional geometries of the brain. In Section C.2, we take some further steps to verify that the reported clearance curves behave as expected with regard to the mesh resolution.

## C.2 Clearance curves under varying mesh resolution and time steps

This section investigates how the clearance curves for the entire rat brain are impacted by varying mesh resolution and the size of time steps used in the simulations. Following the procedure from Section 2.6, we create different meshes of varying resolution. The smallest and largest cell size corresponding to each of the resolutions are listed in Table 8. For each of these meshes, we simulate a pure diffusion model to investigate the impact of mesh resolution on different tracer measurements of interest. Results can be found in Fig 11. We observe a slight difference between the clearance curves obtained from the mesh with resolution 16 and the mesh with resolution 32. However, the clearance curves obtained from the 64- and 32-resolution mesh are virtually indistinguishable. We conclude that our scheme con-verges for the pure diffusion model and the mesh with resolution 32 produces accurate results.

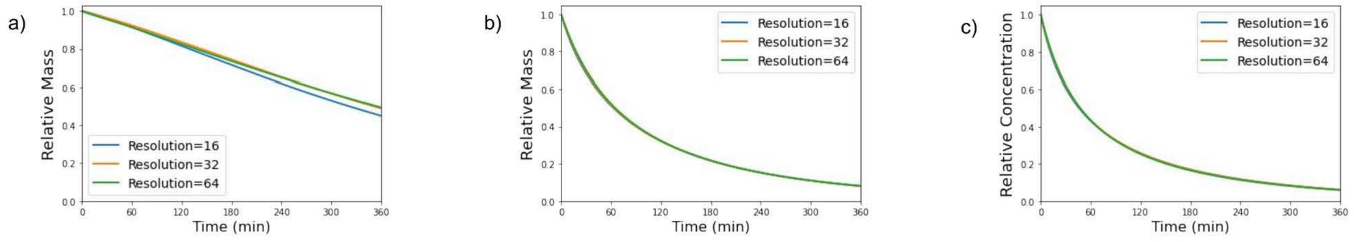


**Fig 11. The evolution of tracer measurements relative to the initial value, plotted for varying mesh resolution.** The simulations were run for a pure diffusion model using the tracer decay model and a time step of 1 minute. a) Relative mass within the entire brain. b) Relative mass within a cube with side lengths of 2mm. c) Relative concentration at the injection point.

**Table 8. The smallest and largest cell size *h* of the mesh for different values of the resolution argument provided to SVMTK.**

| Resolution | $h_{\min}$ | $h_{max}$ |
|---|---|---|
| 16 | 0.265 | 2.374 |
| 32 | 0.154 | 1.190 |
| 64 | 0.073 | 0.622 |

Similarly, we investigate the impact of varying the time step sizes on the clearance curves. The results are shown in Fig 12 and illustrate that a time step of $\delta t = 60$ seconds, as used in our simulations, is sufficiently accurate.

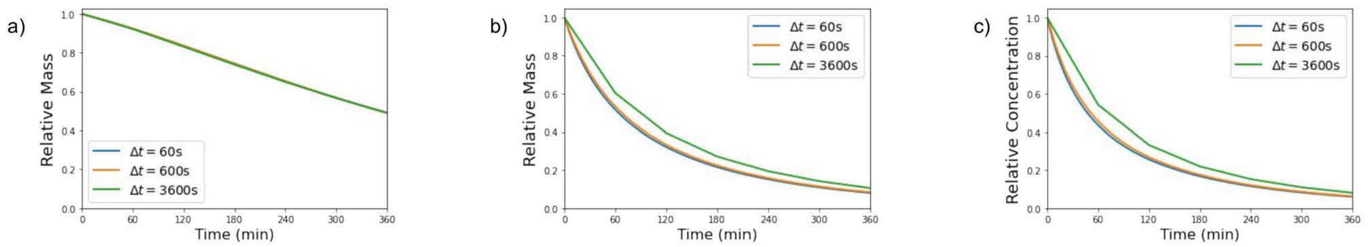


**Fig 12. The evolution of tracer measurements relative to the initial value in the pure diffusion model, plotted for varying time step sizes.** The simulations were done using the tracer decay model and a mesh with resolution 32. a) Relative mass within the entire brain. b) Relative mass within a cube with side lengths of 2mm. c) Relative concentration at the injection point.

Next, we plot the clearance curves for the 7-compartment model for both varying mesh resolutions and time step size in Fig 13. The behaviour for the full model is similar to the pure diffusion model and indicates that further refining the mesh or reducing the time steps will have minimal impact on the clearance curves, especially if we compare it to the uncertainty in other parameters.

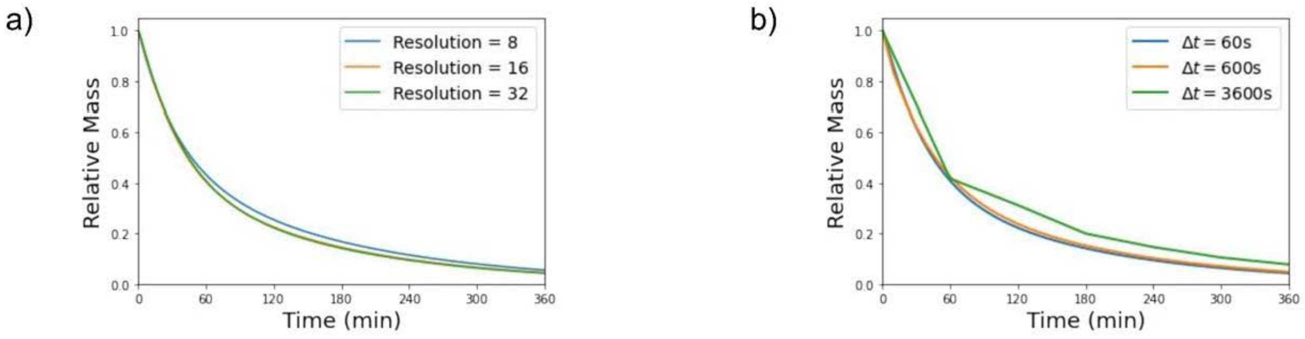


**Fig 13.** The evolution of total tracer mass relative to the initial value in the 7-compartment model, plotted for a) varying mesh resolution (with a timestep of 60s) and b) varying time step sizes (with mesh resolution 32).

Finally, we study the convergence properties of the numerical method for solving pressure equations. We use the solution of the 64-resolution mesh as a reference solution and compute the $L^2$ and $H^1$ error norm with the solutions for the different mesh refinement levels. We obtain the results stated in Table 9 for second-order Lagrange polynomials.

**Table 9. Computed $L^2$ and $H^1$ error norms and convergence orders for our numerical method to solve the pressure equation of the multi-compartment model using second-order Lagrange elements.**

| Resolution | $L^2$-error norm | order | $H^1$-error norm | order |
|---|---|---|---|---|
| 8 | 748 | | 2302 | |
| 16 | 404 | 0.89 | 1659 | 0.47 |
| 32 | 82 | 2.29 | 851 | 0.96 |
